# Monitoring and Behavioral Control of Lepidopteran Pests in Soybean: Evaluation of Food-Based Attractants, Trap Performance, and Selectivity on Non-Target Organisms

**DOI:** 10.1007/s13744-026-01428-0

**Published:** 2026-09-10

**Authors:** Vinicius Cesarin, Sabrina Cesarin de Oliveira, Allana Grecco Guedes, João Rafael Silva Soares, Odair Aparecido Fernandes

**Affiliations:** 1 School of Agricultural and Veterinary Sciences, São Paulo State Univ (UNESP), Jaboticabal, Brazil; 2 Dept of Biology, Faculty of Philosophy, Sciences and Letters, Univ of São Paulo (USP), Ribeirão Preto, Brazil

**Keywords:** Attract-and-kill, Integrated Pest Management, Natural enemies, Scouting, Semiochemicals, Selectivity

## Abstract

Chemically mediated behavioral strategies, including semiochemicals and food-based attractants, have gained prominence in Integrated Pest Management (IPM) programs because of their potential for selective and environmentally friendly pest control. The objective of this study is to evaluate the use of food-based attractants and two trap models (delta-ajar and funnel-ball) for monitoring adult lepidopteran pests in soybean fields. Additionally, the field performance and selectivity of an attract-and-kill (attractant + insecticide) strategy were assessed. Field trials were conducted in commercial soybean areas in Jaboticabal, São Paulo state, Brazil. Results indicated that funnel-ball traps captured a significantly higher number of target moths (36.8 ± 4.3 moths/trap/month) than delta-ajar traps (17.4 ± 2.9 moths/trap/month). However, both models presented similar species richness. Temporal patterns of moth abundance varied along the crop cycle and *Helicoverpa armigera* and *Chrysodeixis includens* populations peaked during the reproductive stages of soybean. In contrast, *Spodoptera frugiperda* and *Elasmopalpus lignosellus* were more abundant during the early vegetative stages, particularly within the first 20 days after plant emergence. The attract-and-kill application was associated with significantly higher moth mortality than the untreated control, especially for *C. includens* and *E. lignosellus*, with mortality peaking at 40 days after trap installation. Importantly, mortality among non-target organisms was relatively low (< 2% of total mortality), and was mainly restricted to a limited number of families such as Dolichopodidae, and Tachinidae (Diptera). These findings underscore the relevance of adaptive, phenology-aware pest management strategies that incorporate semiochemical tools with minimal ecological disruptions.

**Supplementary Information:**

The online version contains supplementary material available at https://doi.org/10.1007/s13744-026-01428-0.

## Introduction

The use of behavior-modifying semiochemicals, including pheromones and plant- or microbe-derived volatiles that function as chemical signals or cues, has gained increasing attention as a selective strategy in Integrated Pest Management (IPM), particularly for insects that rely on chemical information to locate food sources, hosts, or mates (Morrison III et al. 2021). These chemical cues are involved in communication or associated with resource location, and can be classified as pheromones when involved in intraspecific interactions or allelochemicals when mediating interspecific interactions (El-Ghany 2019; Thegusha et al. 2024). The detection of these cues by insect sensory systems triggers essential behaviors, such as foraging, oviposition, and mating, directly influencing population dynamics (Palial & Nidhi 2019). Advances in the synthesis, formulation, and field application of these compounds have broadened their potential in agriculture, allowing targeted behavioral manipulation of pest species with reduced environmental impact. Strategies such as attract-and-kill, mating disruption, mass trapping, and push-pull systems are now well-established components of IPM programs, enhancing control efficacy while preserving non-target organisms (Bakthavatsalam 2016; Shashank et al. 2024).

Sex pheromones are widely used in these approaches. However, their application has some limitations. For instance, many formulations attract only males, which may reduce their effectiveness in species with complex mating behaviors or where fertilized females actively disperse (Fu et al. 2022). Furthermore, pheromones have not yet been identified or made commercially available for all economically important insect pests. In this scenario, food-based attractants offer a promising alternative, as they attract both sexes and are highly compatible with insecticides, making them suitable for use in coupled behavioral and chemical control strategies (Kong et al. 2020). These formulations often rely on volatile compounds derived from plant materials, protein hydrolysates, or fermentation products, which insects exploit as resource-location cues. Plants, for example, release volatiles that insects use to locate food sources and oviposition sites, and such compounds can be incorporated into attractive formulations employed in the field (Gregg et al. 2018; Xu & Turlings 2018). These chemically mediated cues have been successfully applied to monitor several arthropod groups, including Lepidoptera and Diptera, thereby strengthening decision-making processes within IPM frameworks (Judd et al. 2017; Delgado et al. 2022; Batallas & Evenden 2023).

In addition to being effective, food-based attractants may also enhance selectivity, since insect responses to chemical stimuli differ according to their sensory ecology and feeding preferences. This property represents a considerable advantage, particularly in agricultural systems that aim to minimize impacts on non-target organisms, such as pollinators and natural enemies (Gregg et al. 2018). Studies have shown that simple, well-defined attractants tend to lure fewer non-target insects than more complex fermented baits, reducing ecological side effects and improving the precision of pest monitoring (Cha et al. 2015). Nevertheless, when combined with insecticides, as in the attract-and-kill strategy, it is essential to evaluate the potential side effects on non-target organisms. Although this approach generally exhibits satisfactory selectivity under field conditions, particularly during periods of low insect density, its performance depends on several factors, including the chemical formulation, method of application, and mobility of exposed organisms (Spitaler et al. 2014). Accordingly, approaches such as confined lure placement within traps or spatial restriction of treated areas have proven effective in mitigating ecological risks and enhancing selectivity (Szanyi et al. 2024).

Another critical factor influencing the success of food attractant or semiochemical-based strategies is crop phenology. The developmental stage of the plant can directly affect both the attractiveness of lures and the susceptibility of pest populations to control methods. Evidence suggests that floral and food-based lures vary in effectiveness depending on the crop’s growth stage, highlighting the need for temporally tailored approaches (Segers et al. 2023; Abana & Amarasekare 2024). Proper timing of applications is a key element in maximizing the efficacy of behavioral monitoring and control strategies. This is especially important in agricultural systems with successive crop cycles, such as second and third growing seasons that are common in Central Brazil, where crops are often sown in rapid succession. Such practices can lead to the continuous availability of host plants and overlapping pest generations, contributing to the emergence of species such as the *Spodoptera* species (Lepidoptera: Noctuidae) complex as major pests in both soybean and cotton, an issue rarely observed in the past (Parra et al. 2021). In this context, IPM must be planned not by individual crops, but by considering the broader agroecosystem, requiring strategies that account for the temporal dynamics and interspecies interactions across the production landscape.

In addition to the chemical nature of attractants, trap design plays a critical role in the efficiency and selectivity of insect monitoring systems. Structural characteristics such as trap shape, color, entrance configuration, and retention mechanisms can strongly influence insect approach, landing behavior, capture success, and escape probability, particularly in nocturnal Lepidoptera (Whitfield et al. 2019; Sisay et al. 2024). Consequently, evaluating trap performance alongside chemical attractants is essential to disentangle chemical attraction from physical capture efficiency and to ensure that monitoring tools reliably reflect adult activity patterns rather than trap-specific biases.

In this context, systems that combine crops with contrasting architectures and phenologies represent a particularly relevant setting for evaluating chemical cue-based strategies. The MEIOSI system (Interrotational Method of Simultaneous Occurrence), in which one or two sugarcane rows (nursery rows), typically established with pre-sprouted seedlings, are planted in a sugarcane reform area and separated by wider inter-row spaces cultivated with leguminous crops such as soybean or peanut, creates a structurally heterogeneous agroecosystem. After harvesting the intercrop, the original sugarcane rows are mechanically cut and laterally distributed to occupy the adjacent area, completing field establishment. This system increases planting efficiency and alters habitat structure, potentially influencing moth movement, resource availability, and population dynamics (Adati et al. 2011; Rossetto et al. 2022). Such systems pose additional challenges for pest monitoring and control, whereas provide an opportunity to assess the field applicability of food-based attractants under field conditions.

In response to these challenges, the aim of this study was to evaluate the use of a food-based attractant in strategies for monitoring and controlling lepidopteran pests in cropping systems characterized by successive cultivation cycles, where overlapping pest populations and continuous host availability pose challenges for effective IPM. Specifically, the objectives were to (i) compare the efficiency of different trap models in monitoring target soybean lepidopteran pest species; (ii) characterize the mortality of target pest species associated with localized applications of a food attractant combined with insecticide throughout the soybean growing season; and (iii) analyze the effects of this approach on non-target organisms, with emphasis on selectivity and the sustainability of the practice.

## Material and Methods

### Study Area

**Fig. 1:**
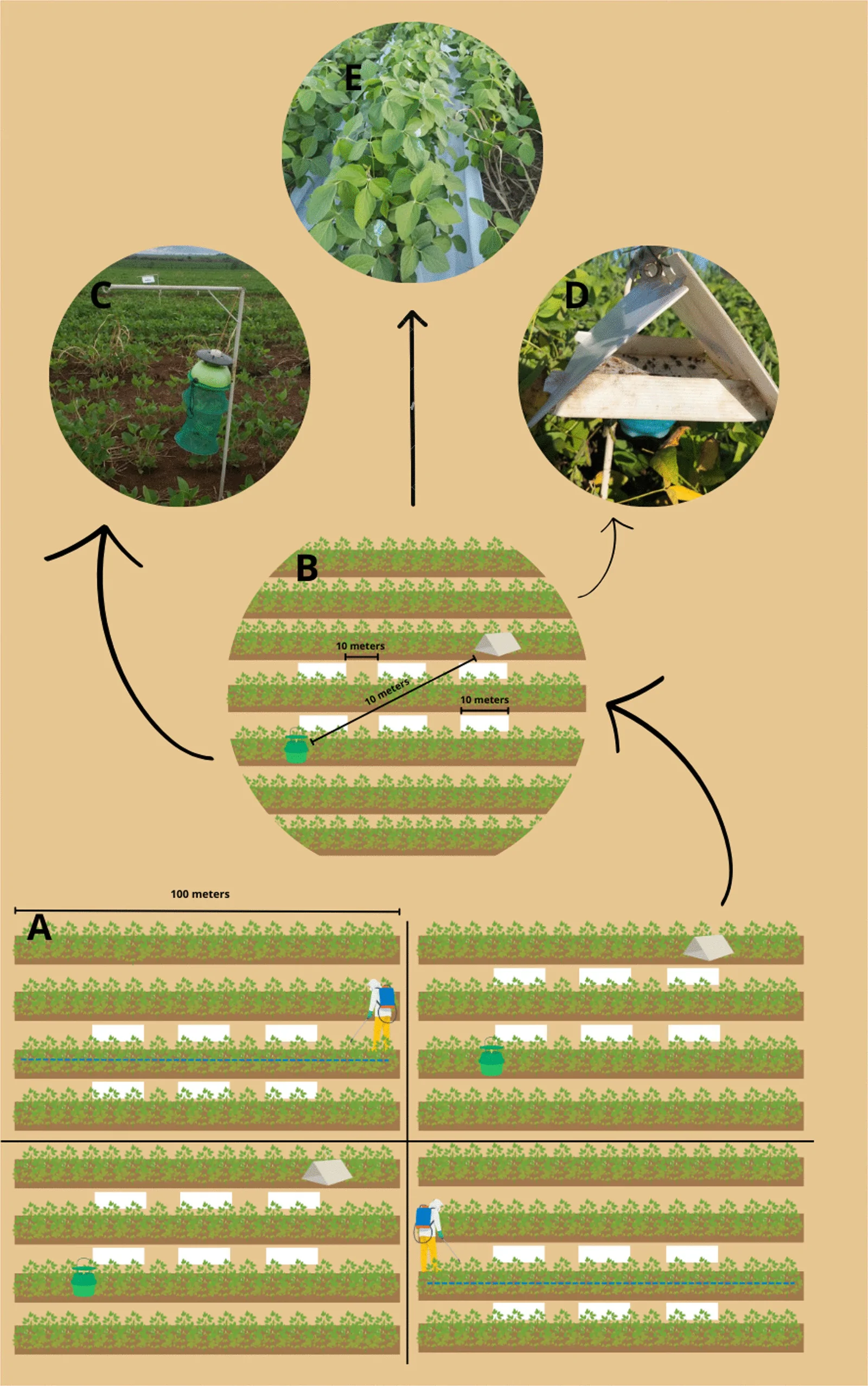
Schematic representation of the experimental design and field setup used for monitoring and controlling lepidopteran pests in soybean. **A** Each experimental block was divided into four 1-ha plots. In two diagonally opposite plots, traps were installed for pest monitoring, whereas in the remaining two plots, insecticide and attractant mixtures were applied when pest thresholds were reached. **B** Details of the plot layout showing the spacing between traps and white fabrics (10 m apart) used to assess insect mortality. **C** Funnel-ball trap and **D** delta-ajar trap installed in the field for monitoring lepidopteran pests. **E** White fabrics were placed next to soybean rows to collect dead insects following insecticide + attractant applications

The experiment was conducted during the 2021/2022 growing season in a commercial soybean field in Jaboticabal, São Paulo state, Brazil. The crop was established under the MEIOSI (Simultaneously Occurring Interrotational Method) system, with sugarcane intercropped with soybean, arranged in a spacing pattern of one sugarcane row for every 27 rows of soybean. Although the MEIOSI system does not represent the dominant soybean production system, its structural complexity provides a relevant context to evaluate the robustness of attractant-based monitoring tools under heterogeneous field conditions. The area was selected based on logistical feasibility, which ensured frequent field visits, standardized trap maintenance, and consistent monitoring throughout the experimental period. Three plots were randomly selected, and within each, four 1-hectare experimental units were delimited, totaling 4 hectares per plot (Fig. 1A). Landscape surrounding the plots was a mix of agriculture grown with sugarcane and natural vegetation. All experimental plots were located in proximity to natural vegetation, although distances varied among sites, ranging from approximately 300 to 700 m from the nearest natural vegetation strip.

In the first two plots, the soybean cultivar BRASMAX FIBRA IPRO (Brasmax Genética, Londrina, PR, Brazil) was used, whereas in the third plot, the cultivar M 6410 IPRO (Monsoy, Campinas, SP, Brazil) was planted. These cultivars are very similar regarding phenological cycle and development. No pest control treatments were applied to the designated experimental areas during the trial. Soybean development was monitored throughout the vegetative and reproductive stages (V1 to R6), according to Purcell et al. (2014).

### Monitoring and Control of Lepidopteran Pests

Lepidopteran pests were monitored using two trap types (delta-ajar and funnel-ball, ISCA Technologies®, Riverside, CA, USA) baited with the food-based attractant Chamariz® (AgBiTech, Campinas, SP, Brazil). According to the manufacturer and previous descriptions of related formulations, this attractant is based on a blend of plant-derived volatile compounds and sugars originally developed for the attraction and control of lepidopteran pests (Gregg et al. 2016a). This formulation includes phenylacetaldehyde, α-pinene, cineole, limonene, butyl salicylate, anisyl alcohol, and sucrose as a feeding stimulant. The exact quantitative composition of the commercial formulation is proprietary and was not fully disclosed by the manufacturer (Gregg et al. 2016a).

These traps were selected based on their proven effectiveness in monitoring lepidopteran pests (Fite et al. 2020; Fleming et al. 2021; Reavey et al. 2025), their widespread use in the study region, and their practical suitability to field conditions. A delta-ajar trap and a funnel-ball trap (Fig. 1C and D) were installed at the centers of two diagonally opposite plots, separated by 10 m. Thus, in each block (replicate), two plots were equipped with traps, while the remaining two served as areas for spraying attractant + insecticide (without traps). The delta-ajar traps were filled with 100 mL of food attractant, and the funnel-ball traps received 25 mL, which corresponds to the holding capacity of the attractant reservoir in both trap types. To prevent access by ants and avoid consumption of captured insects, a band of automotive grease was applied to the base of all trap poles. The traps were installed 20 days after soybean sowing.

Sampling was carried out twice a week, with a minimum interval of 3 days between each sampling, totaling 18 sampling events throughout the study. In some weeks, sampling was performed only once because of weather conditions that prevented access to the field. During each sampling, the captured insects were collected, the adhesive floor of the delta-ajar trap was renewed, and the collection bag of the funnel-ball traps was changed. Although the manufacturer recommends replacement intervals of up to 7 days, the attractant was replaced at each sampling event to minimize variability in lure performance during this initial evaluation. After collecting, the insects were counted and identified. The contents discarded during attractant replacement were poured into a container and disposed of outside the experimental area.

Pest control was implemented based on the infestation level of adult lepidopteran pests in soybean, monitored using traps. Control actions were initiated when an average of 10 adults per trap was recorded for at least two consecutive sampling events. This value was adopted as a standardized operational criterion to trigger attract-and-kill applications and ensure consistency among plots, rather than as a formal economic injury level for soybean. The control consisted of applying a food-based attractant combined with the insecticide methomyl (Bazuka 216 SL®, Rotam Brasil, Campinas, SP, Brazil) at a concentration of 2% c.p. The spray solution was applied exclusively to a single central soybean row within each of the two plots without traps (Fig. 1A), corresponding to a treated row length of approximately 100 m, with soybean rows spaced 0.5 m apart. A total application volume equivalent to 1 L ha⁻^1^ was delivered as a continuous, directed stream (“jet application”) onto the soybean canopy. Applications were performed using a manual backpack sprayer without a spray nozzle, as the high viscosity of the attractant formulation prevented atomization and the use of conventional spray tips. The spray was manually distributed as uniformly as possible along the entire length of the row, ensuring that all plants received the treatment. Methomyl was selected because of its efficacy as a contact and ingestion insecticide with a knock-down effect for lepidopteran pests. Applications were performed in the late afternoon to coincide with the period of increased moth activity, which typically occurs at dusk and early evening (Orlandin et al. 2016).

Over the course of the study, four control applications were carried out, although the third was compromised by rainfall shortly after the spraying. Although the same attractant was used for monitoring and control, traps were installed only in designated plots and spatially separated from treated rows, minimizing potential behavioral interference between lure sources.

### Assessment of Food Attractant + Insecticide Mixture for Lepidopteran Pest Control

To evaluate the field performance of the control tactic and its impact on non-target insects, three pairs of white voile fabrics (10 m long and 0.5 m wide) were placed over the soil alongside the central soybean row that received the application of the food-based attractant combined with the insecticide (Gregg et al. 2016b) (Fig. 1B). Each pair of fabrics was positioned 10 m apart along the crop row (Fig. 1E). The fabrics were also installed in plots where no product was applied (untreated control) to estimate background insect mortality under field conditions. Twelve hours after application, all dead arthropods found on the fabrics were collected and preserved in 70% ethanol for subsequent identification and counting.

This two-treatment design was adopted because the study aimed to test the practical field performance of the complete attract-and-kill formulation, as it would be used operationally, rather than to separate the independent effects of the food attractant and the insecticide. The untreated control was included to provide a baseline for natural mortality and incidental arthropod deposition on the fabrics. Therefore, the comparison allows inference on whether the combined, localized attractant + insecticide application increased observed mortality relative to background mortality, but it does not allow the attractant effect to be isolated from the insecticide effect. A full factorial design including insecticide alone, attractant alone, attractant + insecticide, and an untreated control would be required to quantify the specific contribution of attraction to moth mortality.

### Statistical Analysis

To formally test whether total moth abundance differed between trap types, we fitted a generalized linear mixed model (GLMM) with negative binomial distribution and log-link function. For this analysis, moth counts were summed per trap type and sampling date (DAI). Trap type was included as a fixed effect, while sampling area and trap identity were included as random effects to account for spatial variation and repeated measurements over time. The significance of the trap type effect was assessed using Wald chi-square tests.

The data were organized by species for moths and orders for non-target insects. We applied Pearson’s correlation analysis between each trap type and each taxon to check the similarity of the capture efficiency.

Data on moth captures from the different trap types were analyzed using Generalized Linear Latent Variable Models (GLLVMs). GLLVMs are a powerful extension of generalized linear models (GLMs) that incorporate latent variables to model hidden dependence structures within the data. They are instrumental in dealing with correlated multivariate responses or hierarchical data structures. GLLVMs allow for the simultaneous modeling of the abundance of all moth species, capturing the shared effects of trap type and sampling areas, species-specific differences, unexplained correlations among species through latent variables, and controlling for data dependence by including random effects (Niku et al. 2019). This approach allowed the assessment of whether trap type influenced moth community structure and enabled visualization of the species’ joint response to the trapping method.

In preliminary analyses, *Spodoptera eridania (Stoll) (Lepidoptera: Noctuidae)* was excluded because its occurrence was extremely sparse and restricted to a single trap type (all six individuals were captured in delta-ajar traps, whereas none was captured in funnel-ball traps). This distribution resulted in convergence problems and unstable parameter estimates in the GLLVM. Although *Spodoptera cosmioides (Walker) (Lepidoptera: Noctuidae)* was also rare, it occurred in both trap types and therefore was retained in the analyses. Two GLLVMs were fitted: the first (null model) included only the species count as the response variable, and the second (full model) included trap type (funnel-ball and delta-ajar) and sampling day (as a numeric variable representing soybean phenology) as explanatory variables. These models were used to assess whether the species correlations remained after accounting for the covariates. Residual correlations indicate the extent to which two species co-occur (or not) after controlling for effects such as the number of days after installation (DAI) and trap type (Niku et al. 2019). As the study involved repeated measurements from the same traps over time, the trap ID was included as a random effect. The loadings extracted from the model were used to perform a cluster analysis to identify patterns among species. For this purpose, the k-means algorithm was employed after standardizing the loadings. The optimal number of clusters was determined using the elbow method based on the evaluation of the variance explained as a function of the number of clusters. The identified clusters were then graphically represented in ordination space, allowing visualization of the resulting clustering patterns. The analyses were performed using the “gllvm” package version 2.0.2, with random effects included in the “StudyDesign” argument of the gllvm() function (Niku et al. 2025). The models were fitted using two latent variables and a negative binomial distribution with a log-link function.

Generalized linear mixed models (GLMMs) were used to model the number of moths killed by the attractant + insecticide application. In these models, the number of dead moths found on the fabrics was modeled as a function of treatment (untreated control; attractant + insecticide), moth species, and soybean phenology (DAI – categorical variable), using a negative binomial distribution with a log-link function. A second GLMM model was employed to identify the possible deleterious effects of the insecticide + attractant mixture on non-target organisms. The number of non-target arthropods found dead in the tissues was modeled based on the treatment (untreated control; attractant + insecticide) and soybean phenology (DAI – categorical variable). Because both cases involved repeated measurements on the same plots and areas, we considered the effects of the plots within each area as nested random effects. Model residuals were evaluated using the “DHARMa” package version 0.4.7 (Hartig 2022). When significant differences were detected, Tukey’s HSD test at a 5% significance level was applied to identify pairwise differences. The GLMMs were fitted using the “glmmTMB” package version 1.1.11 (Brooks et al. 2017), and treatment comparisons were performed using the “emmeans” package version 1.10.2 (Lenth 2024). All statistical analyses were conducted in R version 4.2.1 (R Core Team 2022).

## Results

### Moth Capture Dynamics

A total of 5136 Lepidoptera was captured during the study, along with specimens belonging to the orders Diptera, Hemiptera, Coleoptera, Hymenoptera, and Neuroptera. Among the lepidopteran pests, there were individuals of *Anticarsia gemmatalis* Hübner (Lepidoptera: Erebidae), *Spodoptera frugiperda* (J.E. Smith) (Lepidoptera: Noctuidae), *S. eridania*, *S. cosmioides*, *Spodoptera albula* (Walker) (Lepidoptera: Noctuidae), *Helicoverpa armigera* (Hübner) (Lepidoptera: Noctuidae), *Chrysodeixis includens* (Walker) (Lepidoptera: Noctuidae), and *Elasmopalpus lignosellus* (Zeller) (Lepidoptera: Pyralidae) (Table 1). Additional Lepidoptera not considered key soybean pests were captured in substantial numbers. These specimens were included in the total Lepidoptera count but were not identified beyond order level because they were outside the scope of the study, which focused on economically important soybean pests. *Spodoptera* spp. corresponds to specimens assigned to the genus *Spodoptera* for which species-level identification was not possible because diagnostic characters were not sufficiently preserved for reliable identification.

**Table 1:** Total number of insects collected in the two types of traps (funnel-ball and delta-ajar) with attractive food-based bait

Taxon	Funnel-ball trap	Delta-ajar trap
All Lepidoptera	2839	2297
*Anticarsia gemmatalis*	105	19
*Spodoptera frugiperda*	19	36
*Spodoptera cosmioides*	5	1
*Spodoptera albula*	27	37
*Spodoptera eridania*	0	6
*Spodoptera* spp.	51	80
*Helicoverpa armigera*	26	30
*Chrysodeixis includens*	879	608
*Elasmopalpus lignosellus*	16	31
Other Lepidoptera (non pest)	1711	1449
Hymenoptera	235	1422
Diptera	389	8914
Coleoptera	65	243
Hemiptera	168	97
Neuroptera	4	6

**Fig. 2:**
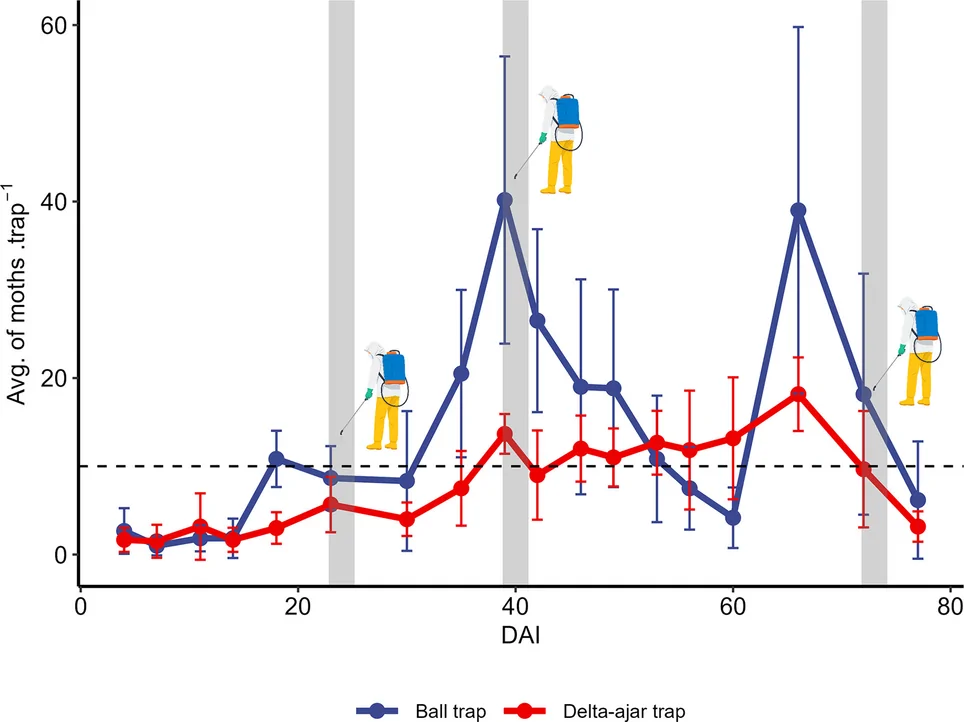
Mean number (± SD) of lepidopteran pests collected in the field using the two trap types (funnel-ball trap and delta-ajar trap) baited with a food-based attractant. The horizontal dashed line represents the control threshold (average of 10 moths per trap), and the shaded gray areas indicate the periods when the attractant + insecticide mixture was applied to soybean rows across DAIs (days after installation). Trap captures were assessed twice per week, with a minimum interval of three days between sampling events

The delta-ajar trap captured a consistently lower number of moths throughout the sampling period compared to the funnel-ball trap (Fig. 2). A generalized linear mixed model with negative binomial distribution indicated a significant effect of trap type on total moth abundance (Wald *χ*^2^ = 8.41, df = 1, *P* < 0.0037), with funnel-ball traps capturing a higher number of moths than delta-ajar traps (Table 2). However, both trap types recorded a similar diversity of lepidopteran species. Additionally, both proved suitable for monitoring lepidopteran pests, displaying similar temporal patterns of increase and decline in the target population (Fig. 2), although trap catches were not compared with an independent estimate of population density; consequently, their ability to provide quantitative estimates of field populations could not be determined.

**Table 2:** Type III Wald chi-square test for negative binomial GLMM adjusted for moth abundance catched by different trap types (bold *P*-values are significant)

Predictors	*χ*^2^	df	*P*-value
Intercept	531.05	1	** < 2.2 × 10**^**–16**^
Trap type	8.44	1	**0.0037**

### Similarity among Moth and Non-Target Organisms Catches Across Different Trap Types

A correlation was observed between the number of insects captured by the traps for all analyzed groups (Table 3), except for the total counts of the orders Hymenoptera, Coleoptera, and Neuroptera and for the species *S. cosmioides*.

**Table 3:** Correlation index between the number of insects collected and the two trap types (funnel-ball and delta-ajar) baited with food-based attractant (bold *P*-values are significant)

Taxon	Correlation (*r*)	*P*-value
Lepidoptera	0.4263	0.0013
All lepidopteran pest species	0.62429	< 0.0001
*Spodoptera* spp.	0.44312	0.0008
*Anticarsia gemmatalis*	0.53307	< 0.0001
*Spodoptera frugiperda*	0.40679	0.0023
*Spodoptera cosmioides*	−0.02879	0.8363
*Spodoptera albula*	0.65776	< 0.0001
*Helicoverpa armigera*	0.48442	0.0002
*Chrysodeixis includens*	0.5809	< 0.0001
*Elasmopalpus lignosellus*	0.49257	0.0002
Hymenoptera	−0.07127	0.6085
Diptera	0.63097	< 0.0001
Coleoptera	0.15725	0.2561

### Effects of Trap Type and Soybean Phenology on Moth Species Composition

The residual correlation matrix among species, derived from the GLLVM full model, considered by trap type (funnel-ball and delta-ajar) and soybean phenology represented by the number of days after trap installation (DAI) as predictors, indicated that interspecific interactions were generally weak, with most correlations being close to zero (Table 4). This low level of co-occurrence suggests that the joint distribution patterns of the species are primarily explained by these two predictors rather than by direct interspecific associations.

**Table 4:** Residual correlation matrix among moth species estimated using GLLVM with negative binomial distribution and predictors DAI and trap type (full model). Values near zero indicate weak residual correlations after adjusting for predictor variables

Species	*Anticarsia gemmatalis*	*Chrysodeixis includens*	*Elasmopalpus lignosellus*	*Helicoverpa armigera*	*Spodoptera albula*	*Spodoptera cosmioides*	*Spodoptera frugiperda*
*Anticarsia gemmatalis*	1.00						
*Chrysodeixis includens*	2.68 × 10^–9^	1.00					
*Elasmopalpus lignosellus*	−4.61 × 10^–10^	−6.53 × 10^–10^	1.00				
*Helicoverpa armigera*	8.06 × 10^–10^	1.14 × 10^–9^	−1.96 × 10^–10^	1.00			
*Spodoptera albula*	2.88 × 10^–10^	4.07 × 10^–10^	−7.00 × 10^–11^	1.22 × 10^–10^	1.00		
*Spodoptera cosmioides*	−6.20 × 10^–11^	−8.77 × 10^–11^	1.51 × 10^–11^	−2.63 × 10^–11^	−9.41 × 10^–12^	1.00	
*Spodoptera frugiperda*	2.31 × 10^–10^	3.27 × 10^–10^	−5.62 × 10^–11^	−9.81 × 10^–11^	3.51 × 10^–11^	−7.55 × 10^–12^	1.00

**Fig. 3:**
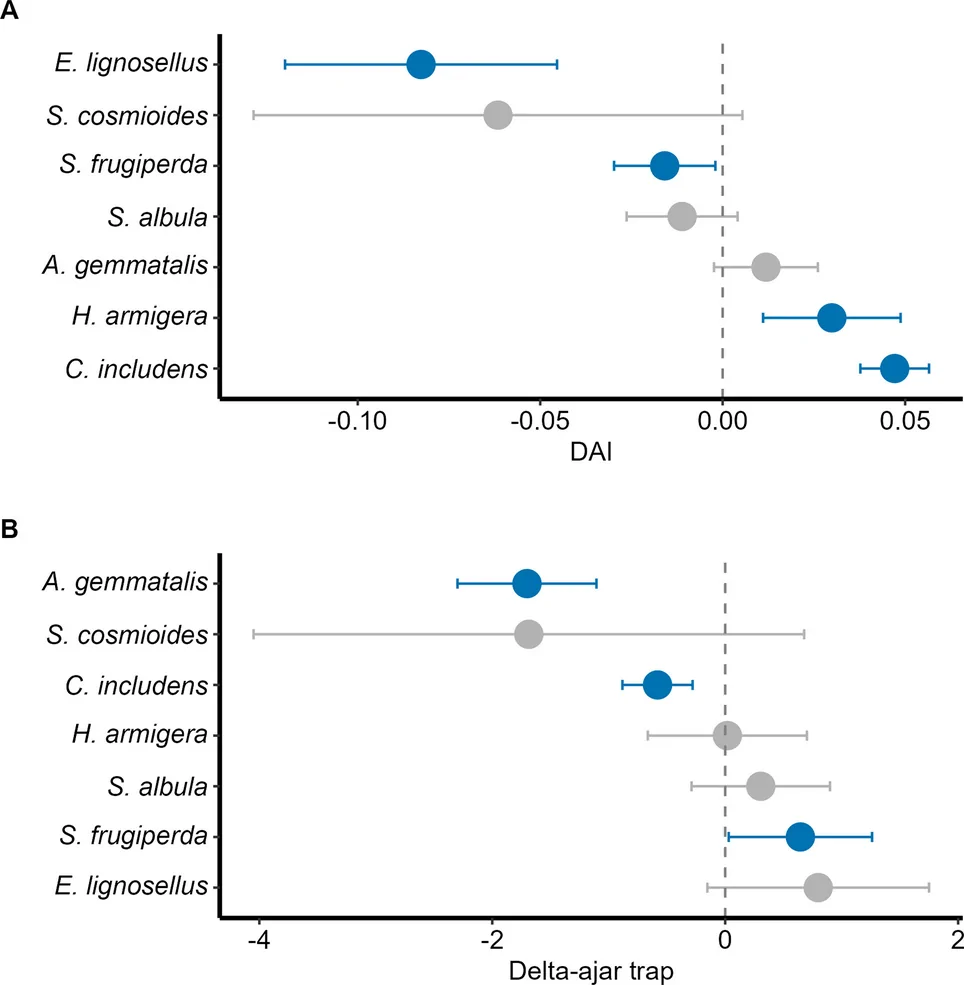
Estimated effects of trap type on the abundance of lepidopteran species based on generalized linear latent variable models (GLLVMs). Points represent coefficients (± CI) for each species: **A** DAI—days after installation—representing soybean phenology; **B** delta-ajar trap, in comparison to funnel-ball trap. Blue points indicate statistically significant effects (*P* < 0.05). Positive values indicated a greater association of species with the DAI or trap type, whereas negative values indicated a lower association

For comparison, an additional analysis using a null model (without predictors) was also conducted and is presented in the supplementary material (Table S1). In this case, slightly higher correlation magnitudes were observed, reflecting raw species co-occurrences before the adjustment of the predictors. The coefficients estimated by the GLLVM indicated that the different trap types influenced species abundance in distinct ways (Fig. 3). Soybean phenology, represented by days after trap installation (DAI; Fig. 3A), had a positive and significant effect on *H. armigera* and *C. includens* (coefficients = 0.03 ± 0.01 and 0.05 ± 0.001, respectively). At the same time, it presented a negative effect on *E. lignosellus* (coefficient = −0.08 ± 0.02) and exhibited a negative trend for *S. frugiperda* (coefficient = −0.02 ± 0.007). The other species did not show statistically significant responses to this predictor variable.

Regarding the delta-ajar traps (Fig. 3B), *A. gemmatalis* was significantly less captured than funnel-ball traps (coefficient = −1.70 ± 0.30), indicating a strong negative response associated with this trap model. *Chrysodeixis includens* (coefficient = −0.58 ± 0.15) also exhibited a significant negative effect, whereas *S. frugiperda* (coefficient = 0.64 ± 0.31) showed a significant positive effect, although both were less pronounced. No significant effects were observed for any of the other species.

**Fig. 4:**
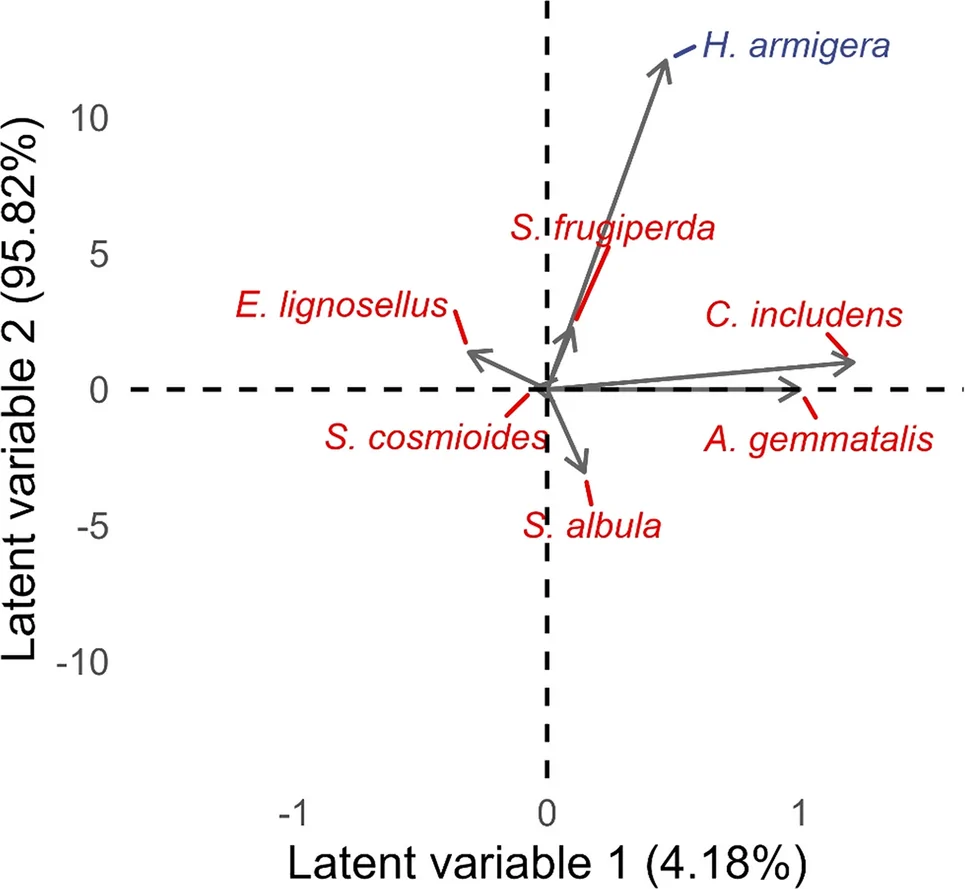
Biplot of lepidopteran species based on the first two latent variables obtained from a generalized latent variable model (GLLVM) using a negative binomial distribution and log-link function. The arrows represent the position of each species in the latent space, reflecting their relationships with the underlying predictor gradients inferred by the model. Colored names indicate species groupings identified by k-means clustering applied to the latent variable loadings. The optimal number of clusters determined using the elbow method was two. Clustering suggests the existence of shared ecological patterns among species, possibly associated with common environmental responses or ecological niches

In addition, the GLLVM revealed clear structural patterns within the moth community observed in soybean fields (Fig. 4). The model with two latent variables (LVs) adequately captured the variation in the data, with the second latent axis (LV2) explaining 95.82% of the structural variance among the samples, whereas the first axis (LV1) accounted for only 4.18%. It is important to note that, unlike principal component analysis (PCA), the latent axes in GLLVM are not necessarily ordered by the proportion of variance explained, as they represent latent dimensions estimated via maximum likelihood.

The ordination of species in the two-dimensional latent space revealed two main clusters, as defined by k-means clustering based on latent coordinates (Fig. 4). One group (in red) included species such as *S. frugiperda*, *C. includens*, *A. gemmatalis*, *S. albula*, *S. cosmioides*, and *E. lignosellus*, whereas the other group (in blue) contained only *H. armigera*.

### Mortality of Lepidopteran Moth Pests Associated with Insecticide + Food Attractant Treatment

**Fig. 5:**
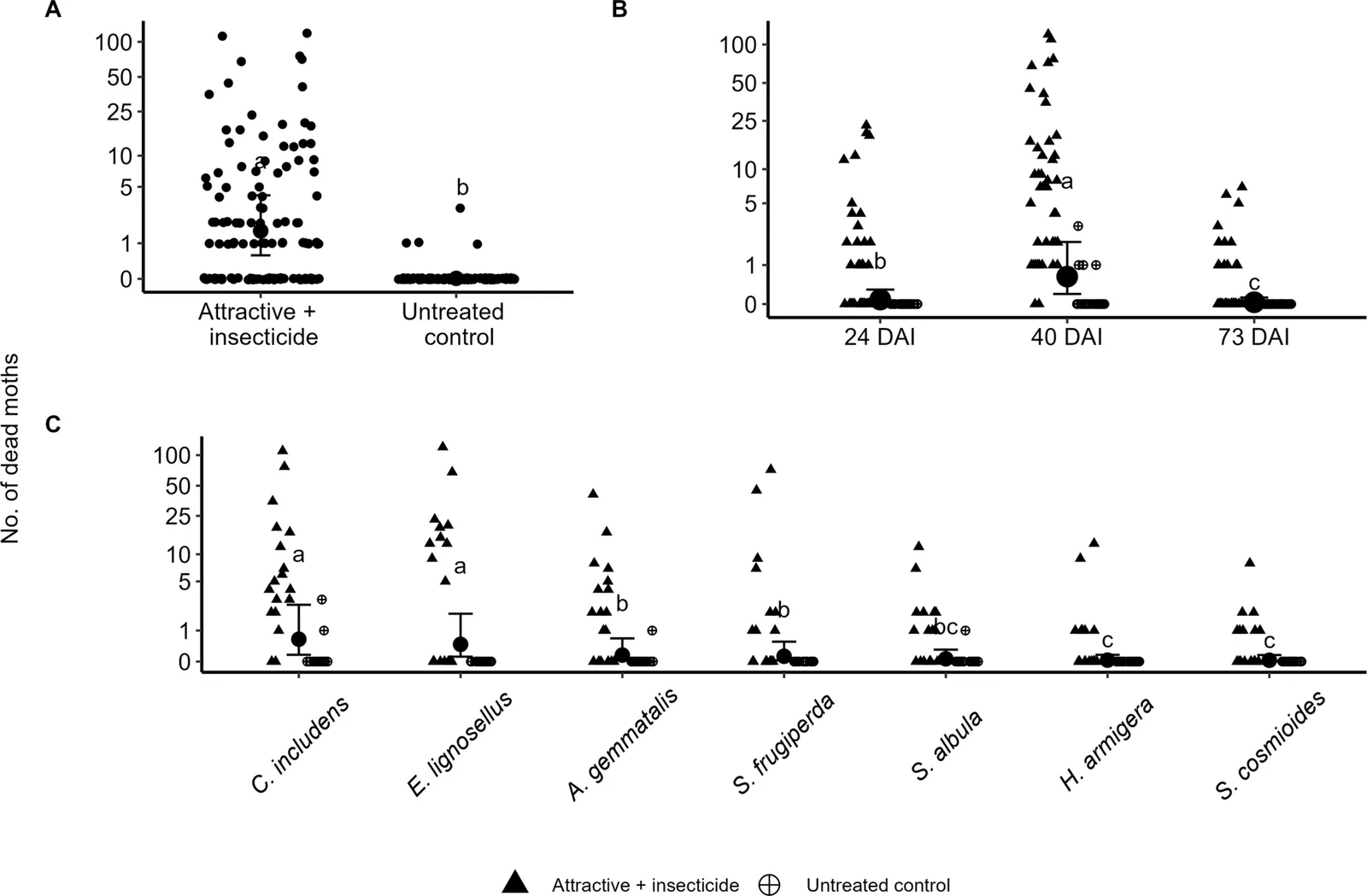
Mortality of agriculturally important moth species following spot application of attractive + insecticide mixture on soybean rows. **A** Overall mortality comparing attractive + insecticide–treated plots and untreated control plots. **B** Mortality over time at 24, 40, and 73 days after installation (DAI). **C** Mortality across the main lepidopteran pest species. Filled triangles represent individual observations from attractive + insecticide–treated plots and cross-circles represent individual observations from untreated control plots. The filled circle with error bars represents the estimated marginal mean ± 95% CI based on a negative binomial mixed model, averaged across treatments. The *y*-axis is displayed on a log(1 + *x*) scale to improve visualization due to high dispersion in raw counts. Different letters indicate statistically significant differences among predicted means (Tukey’s test, *α* = 0.05)

Using a generalized linear mixed model (GLMM) with a negative binomial distribution, we evaluated moth mortality as a function of three explanatory variables: treatment (attractive + insecticide vs. untreated control), moth species, and days after trap installation (DAI). The Type III Wald chi-square test results indicated that all tested variables had a significant effect on moth mortality (Table 5). Insecticide treatment resulted in significantly higher mortality than the control (*χ*^2^ = 114.18, df = 1, *P* = 5.28 × 10^–19^; Fig. 5A). Model-based estimated marginal means indicated that treated plots presented an average mortality of 1.54 individuals per sampling event (95% CI = 0.58–4.09), whereas control plots exhibited a near-zero baseline mortality of 0.009 individuals (95% CI = 0.002–0.039).

**Table 5:** Type III Wald chi-square test for negative binomial GLMM adjusted for moth mortality and non-target insects after spraying the mixture of food attractant + insecticide (bold *P*-values are significant)

Response variable	Predictors	*χ*^2^	df	*P*-value
Moth mortality	Intercept	45.48	1	1.54 × 10^–11^
	Treatment	114.18	2	1.19 × 10^–26^
	Species	98.45	6	5.28 × 10^–19^
	DAI	118.33	1	2.01 × 10^–26^
Non-target insects	Intercept	34.35	1	4.61 × 10^–09^
	Treatment	63.36	1	1.75 × 10^–14^
	Families	300.13	18	4.53 × 10^–53^
	DAI	300.12	2	1.21 × 10^–11^

Soybean phenology also had a significant effect on the number of dead moths (*χ*^2^ = 118.33, df = 2, *P* = 2.01 × 10^–26^; Fig. 5B), with the highest mortality observed at 40 DAI. Model-based estimated marginal means indicated that mortality peaked at 40 DAI, with an average of 0.63 dead moths per sampling event (95% CI = 0.20–2.02), significantly exceeding mortality observed at 24 DAI (0.08; 95% CI = 0.02–0.29) and 73 DAI (0.03; 95% CI = 0.009–0.12). In addition, a significant variation in species-specific mortality was observed (*χ*^2^ = 98.45, df = 6, *P* = 1.19 × 10^–26^; Fig. 5C) Estimated marginal means derived from the GLMM indicated that *C. includens* and *E. lignosellus* presented the highest mortality rates (0.65, 95% CI = 0.16–2.56 and 0.47, 95% CI = 0.11–1.91 individuals per sampling event, respectively), significantly differing from other species. Species such as *H. armigera* and *S. cosmioides* showed significantly lower mortality levels (*H. armigera* = 0.032, 95% CI = 0.0062–0.16; *S. cosmioides* = 0.029, 95% CI = 0.0054–0.16, respectively). Notably, leaves sprayed with the attractant + insecticide mixture exhibited phytotoxicity symptoms (Fig. S1).

### Non-Target Arthropod Mortality Associated with the Insecticide + Food Attractant Treatment

Among the insects collected on the fabrics following application of the insecticide combined with the food-based attractant, less than 2% of the dead individuals belonged to orders considered beneficial, such as Hymenoptera, Neuroptera, and Dermaptera (Table 6). A total of 19 non-target families were recorded in the study area.

**Table 6:** Number and percentage of insects collected on the cloths after the application of insecticide + attractant

Order	1st sampling		2nd sampling		3rd sampling	
	*N*	%	*N*	%	*N*	%
Lepidoptera	287	19.18	2539	76.29	96	31.79
Lepidopteran pests	129	8.62	643	19.32	55	18.21
Diptera	452	30.21	455	13.67	44	14.57
Hymenoptera	7	0.47	18	0.54	4	1.32
Coleoptera	512	34.22	178	5.35	114	37.75
Hemiptera	227	15.17	106	3.19	31	10.26
Blattodea	0	0	2	0.06	0	0
Neuroptera	7	0.47	20	0.60	1	0.33
Orthoptera	1	0.07	9	0.27	11	3.64
Thysanoptera	2	0.13	0	0	0	0
Dermaptera	1	0.07	1	0.03	1	0.33
Total	1496		3328		302	5126

**Fig. 6:**
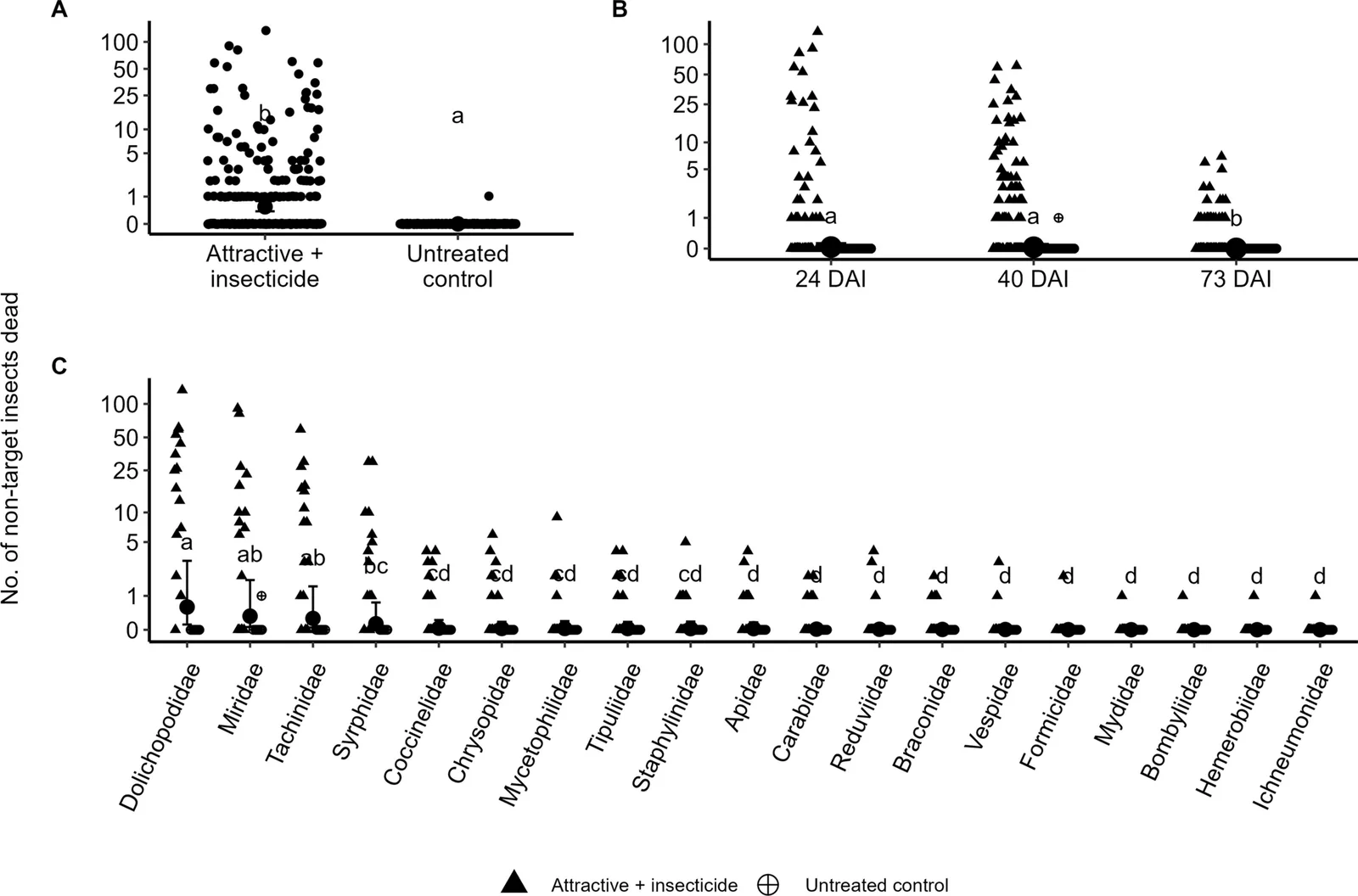
Mortality of non-target insects following spot application of attractive + insecticide mixture on soybean rows. **A** Overall mortality comparing attractive + insecticide–treated plots and untreated control plots. **B** Mortality over time at 24, 40, and 73 days after installation (DAI). **C** Mortality across the main insect families providing potential ecosystem services. Filled triangles represent individual observations from attractive + insecticide–treated plots and cross-circles represent individual observations from untreated control plots. The filled circle with error bars represents the estimated marginal mean ± 95% CI based on a negative binomial mixed model, averaged across treatments. The *y*-axis is displayed on a log(1 + *x*) scale to improve visualization due to high dispersion in raw counts. Different letters indicate statistically significant differences among predicted means (Tukey’s test, *α* = 0.05)

Insecticide treatment significantly increased non-target mortality (*χ*^2^ = 45.96, df = 1, *P* = 1.75 × 10⁻^14^) (Table 5). Model-based estimated marginal means indicated that treated plots presented an average of 0.54 non-target individuals per sampling event (95% CI = 0.38–0.79), whereas control plots exhibited virtually null baseline mortality (0.0005; 95% CI = 0.00005–0.0053) (Fig. 6A). Time after installation (DAI) also significantly influenced non-target mortality (*χ*^2^ = 63.36, df = 2, *P* = 1.21 × 10⁻^11^). Predicted mortality was higher at 24 DAI (0.036; 95% CI = 0.010–0.130) and 40 DAI (0.031; 95% CI = 0.009–0.111), whereas substantially lower mortality was observed at 73 DAI (0.004; 95% CI = 0.001–0.017) (Fig. 6B). Finally, significant differences among non-target insect families were detected (*χ*^2^ = 300.13, df = 18, *P* = 4.53 × 10⁻^53^). Dolichopodidae (Diptera) exhibited the highest predicted mortality (0.59; 95% CI = 0.11–3.09), followed by Miridae (Hemiptera) (0.32; 95% CI = 0.06–1.76) and Tachinidae (Diptera) (0.26; 95% CI = 0.05–1.43) (Fig. 6C). Most other families presented predicted mortality values below 0.05 individuals per sampling event.

## Discussion

Both trap types captured adults of different lepidopteran pest species, reinforcing the potential of food-based attractants as valuable tools for monitoring soybean pests. However, the funnel-ball trap captured significantly more individuals of key pest species, particularly moths from the family Noctuidae and retained insects alive for a few days after capture. This trap also captured fewer beneficial organisms, such as representatives of the orders Hymenoptera and Neuroptera, suggesting greater selectivity, a crucial attribute in strategies aimed at minimizing ecological impact. Similar variation in efficiency among trap models was reported for *H. armigera* in cotton (Lampiri et al. 2025). Thus, the selection of a trap model should consider not only the overall performance but also the behavior and ecology of the target species (Rossini et al. 2025).

Differences in the number of individuals captured may be related to structural and visual characteristics that modulate insect approach, landing, and retention behavior. The conical shape of the funnel-ball trap may facilitate insect entry and hinder escape, as reported for *Pleuroptya ruralis* Scopoli (Lepidoptera: Crambidae) in cone-shaped traps (Shibuya et al. 2023). Its green color may also contribute to attraction of moths, though these insects are generally nocturnal. Studies on *S. frugiperda* have shown that adults, particularly females, prefer darker and green tones and respond to combined visual and olfactory cues (Liu et al. 2024).

Conversely, the white delta-ajar trap potentially attracts a broader diversity of insects, including pollinators and natural enemies. White and translucent traps captured more beneficial hymenopterans, such as *Bombus* spp. (Apidae), as observed by Sircom et al. (2018). These findings indicate that interactions among trap structure, attractants, and insect sensory perception can influence capture patterns under field conditions (Teixeira et al. 2010; Rodriguez-Saona et al. 2020).

Soybean phenology also influenced the abundance of certain lepidopteran species. Captures of *H. armigera* and *C. includens* increased throughout the crop cycle, possibly because flowers and pods represent key sites for adult activity and oviposition (Suzana et al. 2018; Ávila et al. 2024). In contrast, *E. lignosellus* and *S. frugiperda* were captured more frequently during early soybean development. The former is usually associated with newly developing plants whereas the latter may occur during both vegetative and reproductive stages (Silva et al. 2020; Ayala et al. 2024). Therefore, phenology (e.g., onset of reproductive structures and formation of denser canopies in later soybean developmental stages) and associated climatic conditions (e.g., increased rainfall) may determine favorable conditions and periods for pest occurrence and development (Carpane et al. 2022; Ayala et al. 2024). Consequently, these differences in occurrence highlight the importance of initiating monitoring according to crop stage, particularly in systems with successive crops or overlapping host availability (Santos et al. 2017; Carpane et al. 2022).

The GLLVM analysis identified two distinct ecological groupings. *Spodoptera frugiperda*, *A. gemmatalis*, *C. includens*, *S. albula*, *S. cosmioides*, and *E. lignosellus* showed similar responses to the evaluated variables, whereas *H. armigera* displayed a distinct pattern. These groupings may reflect differences in ecological preferences and activity patterns among species, likely associated with the use of distinct ecological niches within the agroecosystem, temporal variation in population dynamics, and varying sensitivity to visual and chemical stimuli provided by the traps (Gerken & Morrison 2023). Low residual correlations after adjusting for covariates further indicated that trap type and soybean phenology explained most variation in captures than direct ecological interactions among taxa (Bahlai et al. 2014; Pachkin et al. 2022). These findings underscore the importance of understanding the ecological structure of insect pest communities in agricultural environments, which enables the development of more targeted monitoring strategies that are sensitive to actual population dynamics and tailored to the specific characteristics of each species.

The food-based attractant combined with methomyl resulted in higher moth mortality than the untreated control, supporting the operational potential of the complete attractant + insecticide formulation under the tested field conditions. The negligible mortality in the control indicated that natural mortality or incidental deposition on the fabrics was low during the 12-h assessment. Responses varied among species being *C. includens* and *E. lignosellus* showed the highest mortality, whereas *H. armigera* and *S. cosmioides* showed lower mortality, possibly because of differences in physiological susceptibility, behavioral exposure to treated droplets, or the degree of response to the food-based attractant (Ríos-Díez & Saldamando-Benjumea 2011; Chen et al. 2019).

Application timing also strongly affected mortality, which peaked at approximately 40 days after trap installation (DAI), coinciding with the maximum moth population activity, and declined sharply by 73 DAI. This result reinforces the importance of precise application timing as a key factor in the success of the attract-and-kill strategy in pest management. When poorly synchronized with pest population dynamics, the application may result in partial or ineffective control, ultimately compromising the overall management strategy (Pinto & Fernandes 2024).

The overall impact on non-target organisms was relatively low, and less than 2% of the insects collected after the application of the attractant + insecticide mixture belonged to taxonomic orders considered beneficial, such as Hymenoptera, Neuroptera, and Dermaptera, indicating a promising level of selectivity. Similar limited effects of plant volatile–based attract-and-kill formulations on generalist predators have been reported in cotton (Gregg et al. 2016a). Nevertheless, high mortality was observed in functionally important families such as Dolichopodidae, Tachinidae (Diptera), and Miridae (Hemiptera), which are known to play relevant roles in natural biological control. The presence of these organisms in traps baited with floral and pheromone lures has also been reported in cotton and soybean fields, although at lower proportions than the target lepidopteran species (Fite et al. 2020). These findings suggest that although the impact on natural enemies was quantitatively low, the direct application of an attractant combined with an insecticide on the crop may negatively affect ecologically sensitive species. Therefore, more selective insecticides and restricted application methods, such as confined-release devices, could further reduce non-target exposure (Rahman & Broughton 2016).

The potential sustainability of this approach derives mainly from the highly localized application of the toxicant. Applying the mixture to a single central row at a low overall application rate (1 L ha⁻^1^) reduced the insecticide load relative to conventional full-area spraying and may limit environmental exposure. Nevertheless, more selective or less toxic active ingredients should be evaluated to improve compatibility with conservation-based IPM programs (Torres & Bueno 2018).

An important limitation is that the two-treatment design (attractant + insecticide and untreated control) cannot separate insecticide-induced mortality from the effect of increased attraction to the treated row. The results therefore indicated the field performance and selectivity of the complete attract-and-kill tactic, rather than the independent contribution of the attractant. Future studies should include insecticide-only and attractant-only treatments in addition to the combined treatment and untreated control to quantify additive or synergistic effects and to determine the specific role of the attractant in increasing pest exposure to the insecticide.

This study was conducted during a single growing season; therefore, its results may not represent interannual variation in climate, crop phenology, and pest pressure. Multi-season studies are needed to assess the stability of the strategy. Because the attractant was replaced at every sampling event to maintain, future evaluations should also determine lure longevity and commercially feasible replacement intervals.

Furthermore, it is also important to note that the soybean cultivar used in this study (M 6410 IPRO) expresses *Bacillus thuringiensis* (Bt) proteins targeting lepidopteran larvae. Although the traps and attract-and-kill strategy evaluated here act primarily on adult stages, reduced larval survival in Bt soybeans may have lowered baseline population pressure, potentially influencing both adult abundance and treatment effects. Although adults were consistently captured, population dynamics and control efficiency may differ under non-Bt conditions. Therefore, further studies in conventional soybean systems are needed.

In summary, food-based attractants used with traps and localized insecticide applications represent a promising strategy for monitoring and controlling lepidopteran pests in soybean monoculture or sugarcane-soybean intercropping systems. Differences among trap models and pest species emphasize the need to consider crop phenology, pest behavior, and application timing. Although the mortality of non-target organisms was relatively low, further refinement of this technology should be aligned with a more integrated and sustainable pest management perspective, capable of balancing pest control efficacy with the conservation of functional biodiversity and reduced reliance on conventional methods.

## Conclusions

This study demonstrated that the use of food-based attractants in combination with different trap models is an effective strategy for monitoring lepidopteran pests and showed promising operational performance for localized control. The funnel-ball trap showed greater efficiency in capturing pest species, particularly those belonging to the family Noctuidae, a result likely associated with the structural and visual features that favor insect retention. The observed mortality associated with the attractant + insecticide application varied among the evaluated species and was influenced by the crop’s phenological stage, proving to be most effective when applied during peak pest population periods. Despite the observed increase in mortality relative to the untreated control, the impact on non-target organisms was generally low, indicating a favorable level of strategy selectivity. These findings suggest that the integration of food attractants into IPM can significantly contribute to the sustainability of agricultural practices, especially when adapted to the ecological particularities of each crop and aligned with the behavioral traits of the target species. Therefore, the adoption of selective traps and the careful use of insecticides are key elements for advancing pest control strategies that are compatible with the conservation of functional biodiversity in agroecosystems.

## Supplementary Information

Below is the link to the electronic supplementary material.

ESM1 (DOCX 17.1 KB)

ESM2 (DOCX 1.19 MB)

## Data Availability

The dataset will be provided upon request.
