# Supplementary material for: Monitoring and Behavioral Control of Lepidopteran Pests in Soybean: Evaluation of Food-Based Attractants, Trap Performance, and Selectivity on Non-Target Organisms
Source: Neotrop Entomol. 2026 Sep 10;55(1):83. doi: 10.1007/s13744-026-01428-0 (PMC13562112; doi:10.1007/s13744-026-01428-0)
Supplement: Supplementary file 1 [file 13744_2026_1428_MOESM1_ESM.docx]

**Table S1.** Residual correlation matrix among moth species estimated using a GLLVM with negative binomial distribution and no predictors (null model). This analysis represents raw co-occurrence patterns among species, without adjustment for environmental or spatial variables

| Species | *Anticarsia gemmatalis* | *Chrysodeixis includens* | *Elasmopalpus lignosellus* | *Helicoverpa armigera* | *Spodoptera albula* | *Spodoptera cosmioides* | *Spodoptera frugiperda* |
| --- | --- | --- | --- | --- | --- | --- | --- |
| *Anticarsia gemmatalis* | 1.00 |  |  |  |  |  |  |
| *Chrysodeixis includens* | 0.059 | 1.00 |  |  |  |  |  |
| *Elasmopalpus lignosellus* | -0.037 | -0.036 | 1.00 |  |  |  |  |
| *Helicoverpa armigera* | 0.030 | 0.027 | -0.019 | 1.00 |  |  |  |
| *Spodoptera albula* | 3.61×10^-3^ | 3.55×10^-3^ | -2.22×10^-3^ | 1.82×10^-3^ | 1.00 |  |  |
| *Spodoptera cosmioides* | -4.48×10^-3^ | -4.40×10^-3^ | 2.75×10^-3^ | -2.26×10^-3^ | -2.71×10^-4^ | 1.00 |  |
| *Spodoptera frugiperda* | 2.26×10^-3^ | 2.22×10^-3^ | -1.39×10^-3^ | -1.14×10^-3^ | -1.36×10^-4^ | -1.69×10^-4^ | 1.00 |
