# Supplementary material for: Monitoring and Behavioral Control of Lepidopteran Pests in Soybean: Evaluation of Food-Based Attractants, Trap Performance, and Selectivity on Non-Target Organisms
Source: Neotrop Entomol. 2026 Sep 10;55(1):83. doi: 10.1007/s13744-026-01428-0 (PMC13562112; doi:10.1007/s13744-026-01428-0)
Supplement: Supplementary file 2 [file 13744_2026_1428_MOESM2_ESM.docx]

**Figure S1** - Phytotoxicity symptoms on soybean plants resulting from the application of insecticide combined with the food-based attractant (A), and a close-up view of leaf injury (B).
